# Effects of a TELephone Counselling Intervention by Pharmacist (TelCIP) on medication adherence, patient beliefs and satisfaction with information for patients starting treatment: study protocol for a cluster randomized controlled trial

**DOI:** 10.1186/1472-6963-14-219

**Published:** 2014-05-15

**Authors:** Marcel J Kooy, Erica CG van Geffen, Eibert R Heerdink, Liset van Dijk, Marcel L Bouvy

**Affiliations:** 1 Department of Pharmacoepidemiology & Clinical Pharmacology, Utrecht Institute for Pharmaceutical Sciences, Utrecht University, P.O. Box 80082, Utrecht 3508 TB, The Netherlands; 2 Netherlands Institute for Health Services Research (NIVEL), P.O. Box 1568, Utrecht 3500, BN The Netherlands

**Keywords:** Interventions, Pharmacists, Telephone, Remote consultation, Patient education, Medication adherence, Patient centered care

## Abstract

**Background:**

Adherence to medication is often low. Pharmacists may improve adherence, but a one-size-fits-all approach will not work: different patients have different needs. Goal of the current study is to assess the effectiveness of a patient-tailored, telephone-based intervention by a pharmacist at the start of pharmacotherapy aimed at improving medication adherence, satisfaction with information and counselling and the beliefs about medicines.

**Methods/Design:**

A cluster randomized controlled intervention trial in 30 Dutch pharmacies, randomly assigned to 1 of 2 intervention groups. Each group consists of an intervention arm and an usual care arm. The intervention arm in the first group is the usual care arm in the second group and vice versa. One intervention arm focuses on patients starting with antidepressants or bisphosphonates and the other on antilipaemic drugs or renin angiotensin system (RAS)-inhibitors. The intervention consists of a telephone call by a pharmacist 2 or 3 weeks after a new prescription. A random sample of pharmacies will send questionnaires 3 months after the first prescription. This contains socio-demographic questions, a measure of beliefs about medicines (BMQ), satisfaction with information received (SIMS, abbreviated) and frequency of pharmacy counselling (Consumer Quality Index, CQI, abbreviated). The primary outcome measure will be medication adherence calculated from dispensing records retrieved 12 months after the intervention. Patients’ beliefs on medication, perception of the quality of information received and pharmacy counselling are secondary outcomes.

**Discussion:**

The TelCIP study will determine the effectiveness of telephone counselling to improve adherence in patients initiating a new treatment. By measuring satisfaction with information and counselling and beliefs about medication the study will also give clues for the reason of a potential increase in adherence. Finally the study will provide information on which patients are most likely to benefit from this intervention.

**Trial registration:**

The trial is registered at http://www.trialregister.nl under the identifier NTR3237.

## Background

Adherence to medication therapy in general is often low [1-3]. Non-adherence to long-term therapies severely compromises the effectiveness of treatment and is therefore critical from both the perspective of quality of life of individual patients and from the perspective of public health and health economics. There are many different factors involved in non-adherence including social and economic factors, the characteristics of the disease and its therapy and health-care provide related factors and patient-related factors such as beliefs about medicines [3-6]. Urquhart et al. and more recently Vrijens et al. argued that three phases of chronic drug treatment can be identified: acceptance of the treatment plan, implementation of the drug regimen and eventually complete discontinuation (non-persistence) of treatment [7,8]. Non-adherence can take place in these three different stages [8].

Non-adherence cannot be regarded as an isolated problem of the patient. The health care provider has to support patients to improve adherence. Patients need information about their medicines to facilitate their appropriate use and understanding of the benefits and risks [5,9,10]. Providing patients with appropriate information about medication has been associated with improved adherence resulting in improved treatment outcomes. In contrast, information not addressing patients’ needs may produce opposite effects [11,12]. A great part of the information provided by the healthcare practitioner is forgotten or remembered incorrectly [13,14]. Therefore it would be desirable to consider repeated opportunities for providing information [15]. But providing information alone is not enough: patients need to be motivated and be involved in decision making [16]. Negative attitudes and barriers that prevent adherent behaviour should be addressed.

Different interventions have been studied to improve adherence. Multidisciplinary and multifactorial interventions were more effective than single focus-interventions.

Ideally interventions should focus on practical and perceptual barriers that affect adherence. Practical barriers may include complex dosage regimens, the size of tablets, the cost of prescriptions, the route of delivery (e.g. rectal or oral) and side effects. In contrast, perceptual barriers are more complex and are based on an internal negotiation between the perceived necessity of the treatment and any concerns relating to it. Interpersonal communication provides opportunity to tailor information to the practical and perceptual barriers of a specific patient [17,18].

Pharmacists can play an important role in improving adherence: they are easily accessible health-care providers, have frequent contacts with patients, have extensive knowledge about drug therapy and are equipped to provide information and monitor patients’ experiences and adherence at visit to the pharmacy. However, it is not always possible to tailor counselling to patient needs [10]. Some patients are unable to visit the pharmacy. Others perceive a lack of privacy in the pharmacy or do not have time for counselling at the moment of the visit. Sometimes patients are already overwhelmed by information provided by other health care providers and therefore not open to receive additional information from the pharmacy.

A different approach might improve patient counselling. Counselling by telephone has proven to be an effective, easy implementable alternative [19,20]. Although it has some disadvantages like the lack of non-verbal communication, it can resolve some of the barriers mentioned above. The patient is counselled in his or her own safe environment and lack of privacy is not an issue. From the health care providers’ perspective: it is easier to implement since the calls can be scheduled. Competent employees can be appointed and can better anticipate on the subject.

Given the above we designed an intervention aimed at preventing patients initiating treatment from becoming non-adherent. We will focus on patient starting with lipid modifying agents, Renin-Angiotensin-System (RAS)-inhibitors, antidepressants or bisphosphonates. We choose these medications because (1) they are intended for long-term use, (2) are prescribed frequently enough to enable the inclusion of a sufficient number of patients during the study period, (3) adherence is often low and (4) the characteristics of patients using antidepressants, bisphosphonates or RAS-inhibitors/lipid lowering drugs are different and patients might weigh risks and benefits of these four groups of medicines differently.

The main objective of the study is to assess the effectiveness of a patient-tailored, telephone intervention by a pharmacist at the start of pharmacotherapy on (1) adherence, (2) beliefs about medicines and (3) satisfaction with information and counselling. We also will assess to what extent counselling by telephone fulfils patients’ needs.

## Methods and design

### Study design

**Figure 1:**
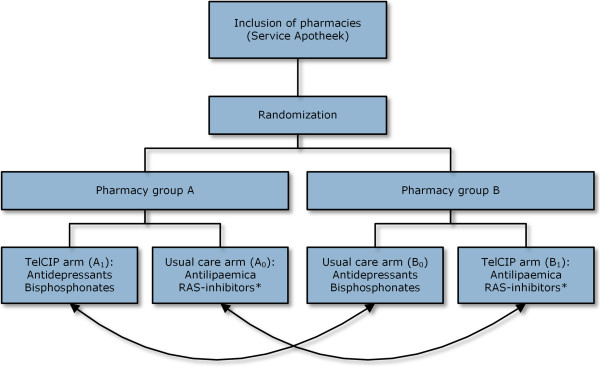
**Study design.** Pharmacies are randomized in two groups. Each group consists of an intervention (TelCIP) arm and a usual care arm. The TelCIP arm in group A focuses on the same medication as the usual care arm in group B and vice versa.

We will conduct a multicentre community pharmacy-based, cluster randomized controlled trial (CRT) (Figure 1). Pharmacies are alternately assigned to either group A or group B in a 1:1 ratio. Given the nature of the study design it is impossible for both the researchers and the pharmacists to be blinded to the group assignment. Each group consists of an intervention (TelCIP) arm and a usual care arm. The TelCIP arm in group A focuses on the same medication as the usual care arm in group B and vice versa.

We performed a pilot in three pharmacies in the period of October 2010 to December 2010. In this pilot we tested the manuals, the feasibility, the software to select the patients (queries) and the online registration form. This pilot led to some practical adjustments in the manuals, the software and the online registration form. The design and the intervention proved to be feasible.

### Recruitment of pharmacies

Independent pharmacies franchisees of ‘Service Apotheek’ are invited to participate in the study. The study design is presented at 4 regional meetings for pharmacies where they could apply for participation. In a weekly newsletter pharmacies are also invited to participate in the study. The pharmacies are periodically visited by staff of the franchise formula and during these visits; the study is also brought to the attention of the pharmacist. Participating health care providers have to follow an e-learning communication training based on the Health Belief Model. The Health Belief Model (HBM) suggests that adherence behaviour is influenced by perceived severity (beliefs about how severe the condition is), perceived susceptibility (the extent to which the patient feels at risk of suffering from the condition) and the effects and disadvantages of the advised behaviour [21,22]. The course aims to train pharmacists and technicians to understand the opinions and behaviour of patients (related to medication intake). Furthermore the training aims to familiarize pharmacists with the concept of concordance. The course also pays attention to sources for information for patients, possibilities and limitations of package leaflet and the package labels. The course takes about three hours, includes case studies and a concluding test to assess the level of theoretical knowledge on communication and concordance.

### Recruitment of patients

Patients starting with treatment will be recruited from 30 community pharmacies in different areas of The Netherlands in the period between May 2011 and March 2013.

Patients in the intervention arm will be selected through an automated selection procedure and presented to the pharmacist. This selection is based on dispensing data and most inclusion and exclusion criteria are incorporated. The same selection will be used to include patients in the usual care group. However not all exclusion criteria can be incorporated in the automated selection and after selection, pharmacist can decide not to include a patient. We will ask the pharmacist to register the reason. However due to the study design the possibility of introducing a selection bias exists, and therefore our primary analysis will be based on the intention-to-treat principle (ITT). Patients will be included in the analysis if they are eligible according to selection criteria based on the pharmacy data. In a per-protocol (PP) analysis we will compare the patients who actually received counselling with patient who received usual care.

#### Inclusion criteria

• Receiving medication for a chronic condition for the first time in 12 months:

• Intervention arm A: starting with an antidepressant or bisphosphonate

• Intervention arm B: starting with a Renin-Angiotensin-System (RAS)-inhibitor or lipid-lowering drug.

#### Exclusion criteria

• Under 18 years of age

• Not responsible for their own medication intake

• Receiving their medication weekly in a multidose dispensing system or multi-compartment dispensing system (e.g. Baxter system or ‘pill organiser’)

• Switching to other medication within the ATC3-group in the 12 months before inclusion

• Receiving medication for a short term indication (e.g. antidepressant for smoking cessation)

• Patients not speaking Dutch nor another language spoken fluently by the healthcare provider

• Patients starting in the same week with both a medication from intervention arm A (antidepressant or bisphosphonate) and a medication from arm B (RAS-inhibitor or lipid-lowering drug).

• Patients without access to a telephone.

Patients in the TelCIP-arms meeting all eligibility criteria receive an information letter, are invited for the study participation and asked for informed consent.

### Medication

The definition of the four different classes of medication is described in detail in Additional file 1. We include antidepressants, bisphosphonates, RAS-inhibitors and lipid lowering drugs. Patients switching within a drug class are excluded. For example when a patient switches from an ACE inhibitor to an Angiotensin II antagonist, the patient is not selected.

### Ethics

The Medical Ethics Review Committee (METC) of the University Medical Centre Utrecht has considered our research proposal in a meeting 13 July 2010 and concluded that the Dutch Medical Research Involving Human Subjects Act (WMO) was not applicable. Consequently the protocol was submitted to the Institutional Review Board (IRB) of UPPER, Utrecht University and they approved the study protocol. The trial was registered at http://www.trialregister.nl under the identifier NTR3237.

### Usual care

Usual care in most Dutch pharmacies is as follows: at the presentation of a first prescription for new medication, the pharmacist or technician provides the patients with spoken and written information about the medication and the disease. Instruction protocols are available and can be used. A first prescription is generally provided for a maximum of two weeks. Guidelines recommend that at the first refill, patients are asked about their experiences with the medication. If necessary, additional information or counselling should be provided. Guidelines for counselling at the first refill, however, are not generally implemented.

### Intervention

The intervention consists of a counselling call by a pharmacist or a competent technician in addition to usual care. The call is supported by a pre-tested interview protocol. For all medication groups a protocol is developed that describes the specific instructions or side effects for that specific group. For example for antidepressants it is mentioned that it can take up to 4–6 weeks to notice an effect. In Additional file 2 a translated version is presented. The focus in the protocol lies on both practical and perceptual barriers to take medication. The need for information about the indication, instructions, side effects and treatment plan will be assessed. Also concerns about the treatment, side effects and dependence will be discussed. The pharmacist will also inquire about the experiences with medication intake during the first 2 weeks of treatment (for example if the patient managed to take the medication, or experienced any possible side effect). The call takes place 7 to 21 days after the first prescription. If necessary the pharmacist will provide information, motivate the patient, help the patient to find a strategy to be adherent or refer the patient to the physician. After the telephone call the pharmacist registers all topics that have been discussed in an online database.

### Follow up

**Figure 2:**
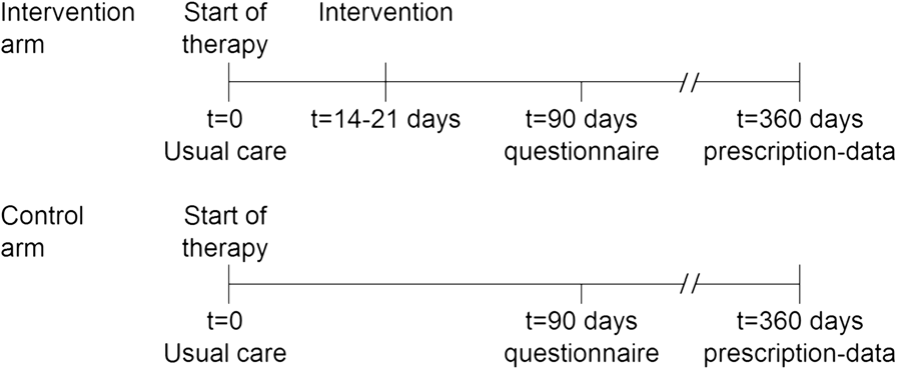
Timeline per patient.

Dispensing data will be extracted from the pharmacy information system. In The Netherlands all prescriptions are registered in an administrative database, including date of prescription, number of prescribed tablets, prescriber and dosage regimen. A selection of pharmacies will collect data on patient beliefs and satisfaction with information and pharmacy counselling through a written questionnaire. In the selected pharmacies a questionnaire will be sent to patients in both arms, three months after the first prescription. The timeline per patient is shown in Figure 2.

### Qualitative analysis of calls

To assess to which extent the pharmacist explores barriers that negatively influence adherence we will record a sample of telephone consultations. These recordings allow a direct analysis of communication without relying on participant reports or simulated situations [23]. In an amendment the Institutional Review Board of the division of Pharmacoepidemiology and Clinical Pharmacology of Utrecht University approved the collection of data.

Patients in the intervention arm meeting all eligibility criteria who give informed consent, are asked for permission to record the consultation.

### Outcomes

#### Primary outcome

The primary outcome is the proportion of adherent patients, based on refill adherence. Refill adherence will be calculated as proportion of days covered over the 360 days following the index date by dividing the total days’ supply by the number of days of study participation (PDC360) [24].

The index date is the date of the first prescription. The total days supplied will be calculated as the sum of days dispensed within the study period. If a supply exceeds the end of the study participation, this supply will be corrected for exceeding the end of the period. The number of days of study participation is defined as the number of days between the index date and the index date + 360 or the last refill date, whichever comes first. For assessing the last refill date, all refills for any drug will be included. We analyse refill adherence both as a continuous measure and as a dichotomous measure with a threshold of 80%. Patients with a PDC360 < 80% are defined as non-adherent and patients with a PDC360 ≥ 80% are defined as adherent.

#### Secondary outcomes

##### Discontinuation

Discontinuation is defined as having a gap of more than 89 days with no medication available within the one year observation period. Cox-proportional hazards will be used to compare discontinuation rates between intervention and control patients.

##### Beliefs about medicines

Patients’ beliefs about medicines will be assessed using the beliefs about medicines questionnaire- specific (BMQs) [25], sent to a random sample of patients three months after the start of therapy. The BMQs assesses both the necessity and concerns regarding prescribed medication. In the questionnaire the name of the specific drug is mentioned in the introduction and wherever it is needed. So for example when a patient starts with simvastatin, one of the BMQ questions will be “I sometimes worry about the long term effects of *simvastatin*”. Five items of the questionnaire assesses the beliefs about the necessity and five items assesses the concerns. Each item of the BMQ is scored using a 5-point Likert scale (1 = strongly disagree, 2 = disagree, 3 = uncertain, 4 = agree, 5 = strongly agree) therefore the individual score ranges from 5 to 25. The results will be expressed as the score on both domains and as the necessity-concerns differential which is the difference between the score on the necessity scale and the concern scale. The results will also be expressed using the two separate scales, divided at the median to generate four attitudinal groups: accepting (high necessity, low concerns), ambivalent (high necessity, high concerns), sceptical (low necessity, high concerns) and indifferent (low necessity, low concerns) [26-28].

##### Satisfaction with information

The satisfaction with information provided by healthcare providers like pharmacists can be assessed with the satisfaction with information about medicines scale (SIMS). As with the BMQ, the name of the specific drug was mentioned in the questionnaire. We used 9 of the 17 items of the original questionnaire [29]. Each item refers to a particular aspect of medicine use. Not all items are used; firstly because some items are not relevant for all four groups of medication, for example “Whether the medication will make you feel drowsy” and “Whether the medication will affect your sex life”. Asking patients these questions when they are not relevant can increase the concerns and thereby influence adherence. Secondly our goal is not to assess the satisfaction in general, but to study the effect of the intervention on satisfaction with information. Thirdly we want to reduce the total number of questions in the questionnaire. We use the items as mentioned in Table 1. We are interested in the effect of the intervention on specific subjects of information and not in an overall satisfaction score. Validation of the combination of the items is therefore not relevant to our study.

**Table 1:** Presentation of selected SIMS items

**Item number**	**SIMS item**	**Original number**
1	How long it will take to act.	5
2	How you can tell if it is working.	6
3	How long you will need to be on your medicine.	7
4	How to get a further supply.	9
5	Whether the medicine has any unwanted effects (side effects)	10
6	What are the risks of you getting side effects.	11
7	What you should do if you experience unwanted side effects	12
8	Whether the medicine interferes with other medicines.	14
9	What you should do if you forget to take a dose.	17
Excluded items:		
	What your medicine is called.	1
	What your medicine is for.	2
	What it does.	3
	How it works.	4
	How to use your medicine.	8
	Whether you can drink alcohol whilst taking this medicine	13
	Whether the medication will make you feel drowsy.	15
	Whether the medication will affect your sex life.	16

Patients are asked to rate the amount of the information received as follows: “too much”, “about right”, “too little”, “none received” and “none needed”. To assess a total satisfaction rating, for each item a score is calculated: if the patient is satisfied (answered “about right”) a score of 1 is given. When the patient is not satisfied (answered “too much”, “too little”, “none needed” or “none received”) this is scored 0. So scores range from 0 to 9, with a high score indicating a high degree of satisfaction. We will calculate a satisfaction score on the same way but based on patients who answered “none received” and “none needed”.

##### Patient’s experience with counselling

The questionnaire contains 4 items adapted from the consumer quality index (CQI) pharmaceutical care [30,31]. In these items the overall experience of different aspects of counselling related to the new medication, is assessed (see Table 2). In the original CQI the patient can answer on a 4-point Likert scale (“never”, “sometimes”, “often”, and “always”). But since we are only interested in the counselling in the first three months since the start of therapy, patients are offered to indicate “yes”, “no” or “I don’t remember”. Patients reporting they received counselling (answered “yes”) will be scored 1 and patients answering “no” or “I don’t remember” will be scored 0. The total score ranges from 0 to 4.

**Table 2:** Frequency of aspects of counselling (adapted from Consumer Quality Index)

**Item number**	
1	Did a pharmacist or pharmacy-employee ask you about your experiences with *the medication*?
2	Did a pharmacist or pharmacy-employee ask you if you suffered from any side effects?
3	Did the pharmacist or pharmacy-employee provide enough personal counselling?
4	Did the pharmacist or pharmacy-employee ask you if you manage to take your medication as prescribed?

#### Other outcomes

All telephone calls and attempts are registered in a database to monitor the implementation in daily practice. For every call or attempt different aspects are registered:

date and duration of the call, number of attempts, age and gender of patient, reasons for not calling the patient

Early discontinuation: did the patient start with the medication or did he/she decide not to start?

Different aspects of knowledge are assessed by the pharmacist on a 5-point scale “Good”, “Sufficient”, “Poor”, “Bad”, “Not discussed”

Experiences and attitude towards medication are assessed by the pharmacist

Advices given during consultation

Contact with prescribing physician in response to consultation.

### Sample size

Power calculation is focused on the primary outcome, the proportion of adherent patients. With a type one error (α) for a two sided test of 0.05 and a probability of rejecting the null hypothesis of 0.80 (1-β) 294 patients per arm are needed for demonstrating an improvement of the proportion of adherent patients from 70% to 80% [32]. For cluster randomisation a correction is needed based on the Intraclass correlation coefficient (ICC). When using ICC = 0.02 we need at least 15 pharmacies to include at least 30 patients per group of medication for the intervention (4), so 15*30*4 = 1800 patients in the intervention arms and 1800 in the usual care arms. We expect an average response rate of 30% on the questionnaires and with the aim to receive at least 100 responses per arm, we estimated to invite at least 670 patients to participate in the survey.

### Statistical analysis

The primary analysis is based on the intention-to-treat (ITT) principle e.g. in the intervention group all patients who should have received the intervention will be included. Patient characteristics between groups will be compared using Student’s t-test or χ^2^- test. Because it is likely that the PDC360 will not be normally distributed, PDC360 differences between groups will be compared using the nonparametric Mann–Whitney U test. We use logistic multilevel analysis to study the effect on the dichotomous primary outcome (adherent yes or no). The outcome of complete discontinuation will be assessed using Cox-proportional hazards. We consider a p-value of less than 0.05 to be statistical significant. In a second analysis effect modification and confounding will be assessed. Effect modification is defined as a significant interaction (p < 0.10) between group allocation and the variable in question. In a per-protocol (PP) analysis we include in the intervention group only patients who actually received the call.

### Handling and storage of data and documents

All patient data will provided to the Utrecht University by the participating pharmacies according to a procedure to protect the subjects’ privacy. Data with regard to the patients’ identity were coded anonymous by the participating pharmacies.

## Discussion

This is the first large intervention trial in The Netherlands to study the effect of telephone counselling by pharmacists on adherence. Although pharmacist can play an important role in improving adherence, in daily practice not all patients receive optimal care. The studied intervention is a way to deliver patient-centred care. And can be a solution to barriers in daily practice and that therefore more patients receive appropriate care. We also recognize that this intervention might not be appropriate for every individual patient, by including sufficient patients in 4 medication groups we expect to gain insight into which patients benefit most of this intervention.

The quality of the intervention depends on the competences and skills of the pharmacist. We try to assure treatment integrity by providing an interview protocol, an obligatory communication training and the obligation to document every counselling-call in an online database. Although it is likely that there will remain some differences between pharmacists, our goal is not to study the effect of an intervention in an ideal, perfectly controlled situation, but to study it in daily practice. We believe that this increases the external validity since it reflects current practice. The qualitative analysis of (a sample of) the telephone calls, will provide more insight in the intervention as provided by different pharmacists.

The intervention focusses on patient starting treatment and the aim of the intervention is to assess both practical and perceptual barriers that can influence adherent behaviour. These barriers can both be intentional or non-intentional and especially at the start of therapy it can be a mix of both. Moreover a recent study suggests that unintentional non-adherence is influenced by medication beliefs, chronic disease and socio-demographics [33]. So before a health care provider can tailor the intervention to intentional or non-intentional non-adherent behaviour, the barriers should first be assessed.

Assessment of adherence will be based on pharmacy data. Studies show that this is a valid method. In the Netherlands most prescriptions are filled for three months, irrespective of the frequency of dosing. Therefore, we expect to find enough contrast to assess the effect of the intervention on refill adherence.

We will conduct this study in different pharmacies in different regions of The Netherlands which will improve the external validity and will make it possible to perform an inter-pharmacy comparison.

### Limitations

The cluster randomized design of the study may compromises the internal validity of the study since difference at baseline between the levels of the provided care between pharmacies cannot be ruled out. It is likely that a part of the patients in the intervention groups will not be available for the intervention, because contact details are lacking or patients cannot be reached. Since in the control group these patients cannot be excluded this can cause a selection bias in the per-protocol analysis.

## Conclusion

Upon completion of this study will have knowledge if and for which group of high-risk patients, counselling by telephone at the start of a pharmacotherapy is (most) effective in improving adherence. Also will be clear how the intervention affects patients’ perceptions on medication and pharmaceutical care.

## Abbreviations

ATC: Anatomical therapeutic chemical; BMQ: Beliefs about medicines questionnaire; CQI: Consumer quality index; HBM: The health belief model; ICC: Intraclass correlation coefficient; ITT: Intention-to-treat; PDC360: Proportion of cays covered of 360 days period; PP: Per-protocol; SIMS: Satisfaction with information about medicines scale; RAS: Renin-angiontensin-system.

## Competing interests

The authors of this protocol disclose no financial competing interest pertinent to this study.

## Authors’ contributions

MK wrote the first draft of the manuscript. MK, MB, KG and RH participated in the design of the trial and study methodology and review of the manuscript. KG, LD, MB and RH made critical revisions to the manuscript. All authors read and approved the final manuscript.

## Authors’ information

Prof. Dr. M.L. Bouvy has been involved in several projects both aiming to describe the magnitude of non-adherence, to explore reasons for patients non-adherence and to improve medication adherence. Moreover M. Bouvy has performed several intervention studies in community pharmacy and is familiar with the practical problems of practice research. M. Bouvy has extended experience with general public information about pharmacotherapy (Books for general public: ‘Drugs in The Netherlands’, ‘The correct drug’ and ‘treat minor ailments’ in cooperation with the Consumers Board). M. Bouvy has frequently given lectures and post graduate courses on medication adherence.

Dr. E.R. Heerdink is associate professor of clinical pharmacoepidemiology at the division of Pharmacoepidemiology and Pharmacotherapy of Utrecht University. He has ample experience with designing, conducting and reporting large-scale studies on the effects of the use of medication in patients in daily clinical practice. He has published extensively in international peer-reviewed journals (150+). Adherence with medication has been a continuous subject of interest throughout his career. He is the 2011/2012 president of the European Society for Patient Adherence, Compliance and Persistence.

Dr. E.C.G. van Geffen is involved in the pharmacy practice research of UPPER, which is part of the division of Pharmacoepidemiology and Pharmacotherapy of Utrecht University. The research mainly focuses on drug induced problems and adherence, patient communication and education, and patients experiences with medication use. Through the UPPER-pharmacy research network, they have access to 900 pharmacies and their patients. Van Geffen finished her thesis on patients’ perspectives and experiences with antidepressant medication. From 1999 to 2008 she was also head of the Science Shop for Medicines of Utrecht University. In 2011 Katja joined the Dutch Kidney Foundation as Program Manager working on the early detection of kidney damage and prevention of kidney failure.

Dr. Ir Liset van Dijk obtained her PhD-degree in sociology at Utrecht University. She was a postdoc at the University of Arizona, the University of Michigan, Utrecht University and Wageningen University. From 1999 onwards Liset is employed at NIVEL, Netherlands institute for health services research in Utrecht, where she is research coordinator of pharmaceutical care. In 2007, she joined the Division of Pharmacoepidemiology and Pharmacotherapy at Utrecht University as adjunct faculty. Her main research interests include: drug utilization research, adherence to medication, international comparisons, and policy evaluation.

M.J.Kooy, PharmD, is a pharmacist combining his care for patients in a community pharmacy with his scientific ambitions at Utrecht University. Since his graduation in 2003 at the University of Groningen, he works in a modern community pharmacy, Service Apotheek Koning in Amsterdam. In 2010 he started working on his PhD thesis on the effects of interventions to improve medication adherence. The main intervention study uses counseling by telephone at the start at therapy as an intervention to improve adherence. He is also involved in two different studies using an electronic reminder device and e-health.

## Pre-publication history

The pre-publication history for this paper can be accessed here:

http://www.biomedcentral.com/1472-6963/14/219/prepub

## Supplementary Material

Additional file 1 **List of included medication.** The table shows the name and ATC-code of the included medication. Click here for file

Additional file 2 **General interview protocol.** The general interview protocol to be used in the counselling calls. Click here for file
