# Supplementary material for: Effects of a TELephone Counselling Intervention by Pharmacist (TelCIP) on medication adherence, patient beliefs and satisfaction with information for patients starting treatment: study protocol for a cluster randomized controlled trial
Source: BMC Health Serv Res. 2014 May 15;14:219. doi: 10.1186/1472-6963-14-219 (PMC4050986; doi:10.1186/1472-6963-14-219)
Supplement: Additional file 1 — List of included medication. The table shows the name and ATC-code of the included medication. [file 1472-6963-14-219-S1.pdf]

RAS-inhibitors	
ATC-code	Name
C09AA01	captopril
C09AA02	enalapril
C09AA03	lisinopril
C09AA04	perindopril
C09AA05	ramipril
C09AA06	quinapril
C09AA07	benazepril
C09AA08	cilazapril
C09AA09	fosinopril
C09AA10	trandolapril
C09AA15	zofenopril
C09BA01	captopril and diuretics
C09BA02	enalapril and diuretics
C09BA03	lisinopril and diuretics
C09BA04	perindopril and diuretics
C09BA05	ramipril and diuretics
C09BA06	quinapril and diuretics
C09BA09	fosinoril and diuretics
C09BB02	enalapril and lercanidipine
C09BB04	perindopril and amlodipine
C09BB10	trandolapril and verapamil
C09CA01	losartan
C09CA02	eprosartan
C09CA03	valsartan
C09CA04	irbesartan
C09CA06	candesartan
C09CA07	telmisartan
C09CA08	olmesartan
C09DA01	losartan and diuretics
C09DA02	eprosartan and diuretics
C09DA03	valsartan and diuretics
C09DA04	irbesartan and diuretics
C09DA06	candesartan and diuretics
C09DA07	telmisartan and diuretics
C09DA08	olmesartan medoxomil and diuretics
C09DB01	valsartan and amlodipine
C09DB02	olmesartan medoxomil and amlodipine
C09DX01	valsartan, amlodipine and hydrochlorothiazide
C09DX03	olmesartan medoxomil, amlodipine and hydrochlorothiazide
C09XA02	aliskiren
C09XA52	aliskiren and hydrochlorothiazide

Lipid lowering drugs	
ATC-code	Name
C10AA01	simvastatin
C10AA03	pravastatin
C10AA04	fluvastatin
C10AA05	atorvastatin
C10AA06	cerivastatin
C10AA07	rosuvastatin
C10AB02	bezafibrate
C10AB04	gemfibrozil
C10AB08	ciprofibrate
C10AC01	colestyramine
C10AC02	colestipol
C10AC04	colesevelam
C10AD02	nicotinic acid
C10AD06	acipimox
C10AX06	omega-3-triglycerides incl. other esters and acids
C10AX09	ezetimibe
C10BA02	simvastatin and ezetimibe

Bisphosphonates	
ATC-code	Name
M05BA01	etidronic acid
M05BA02	clodronic acid
M05BA03	pamidronic acid
M05BA04	alendronic acid
M05BA06	ibandronic acid
M05BA07	risedronic acid
M05BB01	etidronic acid and calcium, sequential
M05BB02	risedronic acid and calcium, sequential
M05BB03	alendronic acid and colecalciferol
M05BB05	alendronic acid, calcium and colecalciferol, sequential
M05BX03	strontium ranelate

Antidepressants	
ATC-code	Name
N06AB03	fluoxetine
N06AB04	citalopram
N06AB05	paroxetine
N06AB06	sertraline
N06AB08	fluvoxamine
N06AB10	escitalopram
N06AX05	trazodone
N06AX11	mirtazapine
N06AX16	venlafaxine
