# Supplementary material for: Effects of a TELephone Counselling Intervention by Pharmacist (TelCIP) on medication adherence, patient beliefs and satisfaction with information for patients starting treatment: study protocol for a cluster randomized controlled trial
Source: BMC Health Serv Res. 2014 May 15;14:219. doi: 10.1186/1472-6963-14-219 (PMC4050986; doi:10.1186/1472-6963-14-219)
Supplement: Additional file 2 — General interview protocol. The general interview protocol to be used in the counselling calls. [file 1472-6963-14-219-S2.pdf]

Interviewprotocol - generic

Space for a label with the address of the patient

Call attempt	Date:	Start time:	End time:	Employee
1				
2				
3				

Patient number: ______

Introduction

Good afternoon Madam/Sir, you speak with [name] of [name of pharmacy]. Around [date] you received from us for the first time [name medicine]. As an extra service we call clients, who recently started with a medicine, ask about your experience with the use of the medicine. Do you have some time?

- ❑ No → *"Can I call you at another moment?"*
  - ❑ Yes. Note date and preferred time on the protocol
  - ❑ No. *"May I ask why not?"…. "Thank you for your time. If you have any questions, you can always call me or visit the pharmacy".*
    Record the reason on the website
- ❑ Yes, I have some time at this moment

→ Explain the (goal) of the study and ask for informed consent.
Does the patient consent? If yes, then continue with the protocol. If no:" *Thank you for your time. If you have any questions, you can always call me or visit the pharmacy".*

Directions for the call:

1. *What's the reason that the physician prescribed this drug?*
   Objective: strike up a conversation with someone with a relative simply question. Identify whether the indication/reason for prescribing is known.

	Good	Sufficient	Moderate	Poor	Not discussed
**Knowledge of indication/reason**	❑	❑	❑	❑	❑

2. *Have you started with [name medicine]?*
   If the patient has not started: identify the reason. Pay attention to resistance to the use of medications specifically and in general.
   If the patient has stopped: ask the reason:______________________

3. *What is your experience so far? How is it going?*
   Objective: identify experiences, motivation and attitude. Pay attention to the motivation. Pay also attention to "doubtful language". Try to find out whether there are any obstacles to not use the medicine. Ask more if something is not clear. Don't try to come directly with solutions and answers. Let the patient formulate the objective of the therapy. Pay attention not only to side effects, but also practical issues such as halving tablets.

4. *What has been told about this medicine?*
   Objective: inventory the nature and scope of knowledge. Pay attention to the next aspect: directly asking the knowledge of the patient can be seen as an exam. When you suspect a gap in one's knowledge or incorrect knowledge: *What do you want to know about this medicine? Shall I tell you something about […gap…]?* If you give some explanation ask if it is sufficient and/or if there are more questions.
   Background information:
   Statins (cholesterol synthesis inhibitors) inhibits the production of cholesterol in the liver and reduce the amount of cholesterol- and fat in the blood. **Ezetimib** inhibits the absorption of cholesterol in the body. Use the medicine continuously. The effect of the **statins** reaches the maximum after 4-6 weeks

   RAS-inhibitors lower blood pressure and improve the output of the heart. Use the medicine continuously. The effect of the **RAS-inhibitors** reaches the maximum after 3-6 weeks

   Bisphosphonates bind to calcium in the bones and inhibit the demolition of the bone. By combining it with sufficient calcium and vitamin D, your bones become stronger. You will not notice the effect of the medicine. But the risk of bone fractures will become smaller. Physicians prescribe this for treatment of bone loss or to prevent it. Use the medicine continuously.

   SSRI's, mirtazepin, venlafaxine help to bring balance to some important substances in the brains. They work best in combination with a physician or psychiatrist. The medication improves the complaints, but cannot take away the cause. Use the medicine continuously. Mostly they start working after 2-4 weeks.

For elderly sometimes even after 6 weeks.

For more information about the side effects: see below:

	Good	Sufficient	Moderate	Poor	Not discussed
**Knowledge about working**	❑	❑	❑	❑	❑
**Knowledge after how much time the medicine works**	❑	❑	❑	❑	❑
**Knowledge about the side effects**	❑	❑	❑	❑	❑

5. *What do you think of getting this medicine? What's your view?*
Objective: access motivation and attitude and incorrect knowledge. What is for the patient the objective to use the medicine?
Alternative formulations: *Do you think that it is important to use this medicine? Are you worried about the side effects in the long term?*

Answer the following statements as a result of the conversation:	Totally agree	Agree	Agree/ disagree	Disagree	Totally disagree	Not discussed
**The patient doubts about the necessity**	❑	❑	❑	❑	❑	❑
**Patient is hesitant for side effects**	❑	❑	❑	❑	❑	❑
**Patient is hesitant for dependence**	❑	❑	❑	❑	❑	❑

6. *How long will you have to use the medicine?*
Objective: is it clear how long the patient has to use the medicine?
Pay attention to the next aspect: try to identify if there is some possible resistance present. *"What's your view on it?"*
If necessary: tell the patients that they always can contact the doctor or pharmacist.

7. Do you know how you can get a refill?

8. *Are you suffering from the medicine?*
Objective: identify if the patient experienced side effects. If someone doesn't have side effects: if necessary continue with asking if it is clear which side effects can appear. Do this to prevent that a side effect will not be recognized.
Additional information:
Side effect **statins**: muscle pain, joint pain, muscle weakness, muscle cramping.
Gastrointestinal complaints, especially in the beginning. If you use the medicine with food, then you can prevent this.
Bisphosphonates:
Sometimes: irritated gullet. Take mediation with full glass of water (no milk) while standing up or sitting up right. After intake, stay 30 minutes up right to prevent that the tablet sticks to the gullet. If you get any pain behind the sternum (chest), contact your physician.
Rare: gastro-intestinal problems, headache, joint pain
Very rare: loss of hare, reduction of vision, pain in jaw, pain in groin, thigh or hip.

SSRI's: most of the time side effects like gastro-intestinal problems, headache, reduced libido, agitation (nervous feeling, distress, confusion) or trembling will fade within 1-2 weeks.
Mirtazepin and venlafaxin: More than 10% of the patients: headache, dry mouth, nausea and sweating 1-10%: vomiting, diarrhea, lethargy, fatigue, dizziness, trembling, strange dreams, distress, insomnia, muscle ache, joint ache, back ache, orthostatic hypotension and exanthema

	Totally agree	Agree	Agree/ disagree	Disagree	Totally disagree	Not discussed
**Patient experienced side effects**	❑	❑	❑	❑	❑	❑

9. *How often do you use the medicines?*
Objective: Try to find out if the patient has problems to use the medicine daily/weekly.
Please prevent to be judgmental. Support the patient in self proposing solutions.
Continue with asking: *"Are you able to use the tablet …time(s) a day/week?"*
Forgotten? **Statins/RAS-inhibitors**: If there are more than eight hours left before taking the next medicine and you use the medicine only once a day, then you can better use the medicine. If there are less than eight hours left, then you can skip it.

10. *When (at what time/moment) do you use the tablets?*
Background: see prescription label. Details:
Statins: Atorvastatin, rosuvastatin, ezetemib: every moment of the day. Other statins: in the evening. In the evening is the production of cholesterol by the liver the highest. At this way you have the most benefit.
Fluvoxamin: preferably 1 dose in the evening. For adults using more than 150mg per day, divide it in to 2-3 gifts per day. For children and adolescents using more than 50mg per day, divide it in to 2 gifts. If different doses per day, take the highest before going to sleep.
Mirtazepin: preferably 1 dose in the evening or when 2 doses are needed: take one in the morning and one in the evening and take the highest dose in the evening
Paroxetin: preferably in the morning
Bisphosphonates: Take the tablet in the morning, directly after getting up. After half an hour you can take your breakfast. This will reduce the effect of food on the absorption.

	Good	Sufficient	Moderate	Poor	Not discussed
**Knowledge about time to use medication**	❑	❑	❑	❑	❑

11. *What are your expectations concerning the use of your medication? Are there any obstacles for you? How can I help you with this?* You can think to help for example with a compliance card, schedule when to use a medicine or week delivery.

Finish

❑ *Repeat eventually agreements. Summarize the conversation.*
❑ *Do you have more questions?*
❑ *Is it ok if I send you every quarterly a newsletter about [name subject]?* **Write the answer on the survey.**
❑ *This pharmacy is working on a study by the University Utrecht on information provided by the pharmacy. Can we send you after around 3 months a written survey?* **Write the answer on this protocol**

Thank you for your time. If you have any questions, you can call the pharmacy or come over in the pharmacy.

Register the counselling online

Additional questions about the consultation

12. It is possible that the patient has made, with or without a consultation with the doctor, changes in the use of the medication. Can you specify whether the following changes have been made:

	Yes	No	Unknown
In consultation with a doctor, the drug is replaced by another drug.	❑	❑	❑
In consultation with the doctor, the patient has stopped the therapy and there is no alternative start.	❑	❑	❑
Without consulting the doctor, the patient discontinued with the therapy.	❑	❑	❑
The patient has changed the daily use without a consultation.	❑	❑	❑

13. What advice have you given *during* the consultation? (Multiple answers possible)
    - ❑ Advice related to the intake of the medicine
    - ❑ Explanation of the duration of the therapy
    - ❑ Explanation/motivation to the indication
    - ❑ Explanation of possible side effects
    - ❑ Explanation possible dependence
    - ❑ Other, ________________

14. Which intervention is in response to the consultation? (Multiple answers possible)
    - ❑ Pilltimer provided
    - ❑ Drug intake schedule provide
    - ❑ Sign up for chronic medication service
    - ❑ Sign up for weekly dose system
    - ❑ Other, ________________
